# Supplementary material for: Single Rh Adatoms Stabilized on α-Fe2O3(11̅02) by Coadsorbed Water
Source: ACS Energy Lett. 2021 Dec 22;7(1):375–80. doi: 10.1021/acsenergylett.1c02405 (PMC8762699; doi:10.1021/acsenergylett.1c02405)
Supplement: Supplementary file 1 — nz1c02405_si_001.pdf [file nz1c02405_si_001.pdf]

Supporting Information

Single Rh adatoms stabilized on α-$Fe_2O_3(1\bar{1}02)$ by co-adsorbed water

Florian Kraushofer[1], Lena Haager[1], Moritz Eder[2], Ali Rafsanjani-Abbasi[1], Zdeněk Jakub[1], Giada Franceschi[1], Michele Riva[1], Matthias Meier[1], Michael Schmid[1], Ulrike Diebold[1], Gareth S. Parkinson[1]

[1] Institute of Applied Physics, TU Wien, Wiedner Hauptstraße 8-10/E134, 1040 Wien, Austria

[2] Chair of Physical Chemistry & Catalysis Research Center, Technical University of Munich, Lichtenbergstraße 4, 85748 Garching, Germany

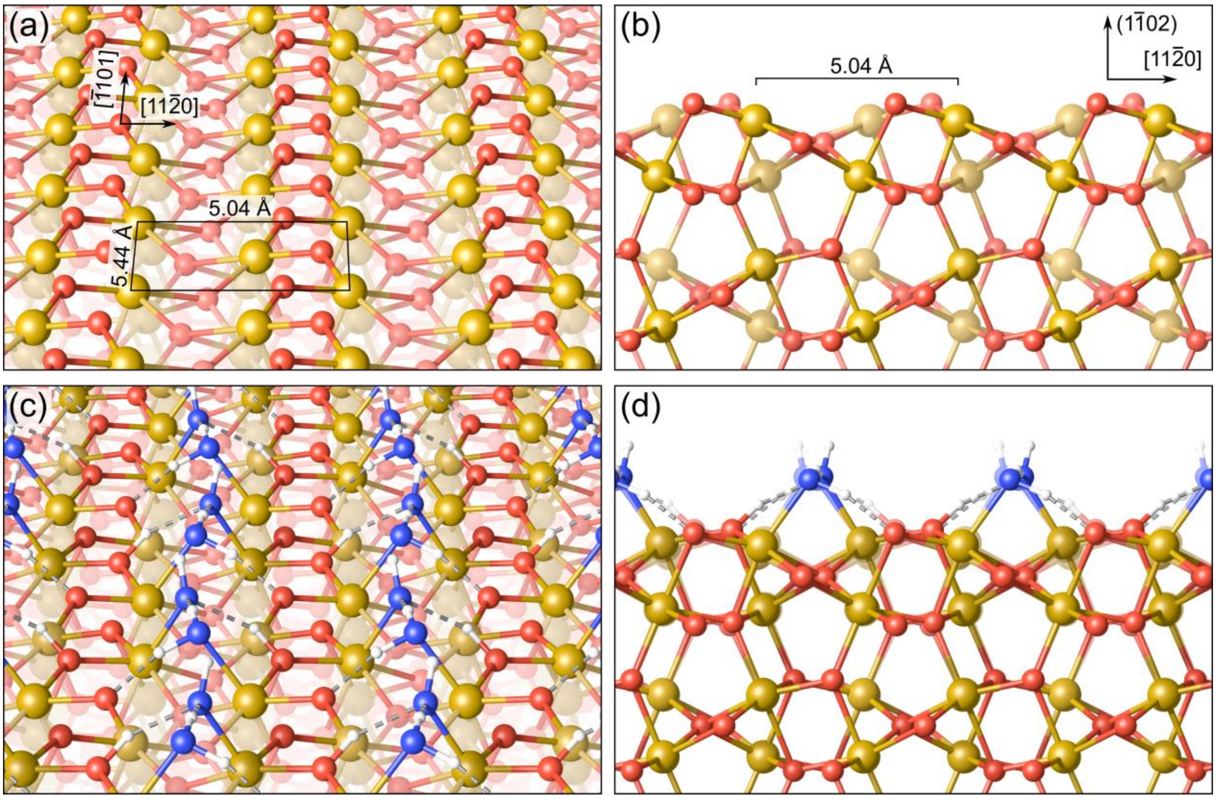

Figure S1: (a, b) perspective and side view of the α-Fe_2O_3(1$\bar{1}$02)-(1×1) termination.[1] Oxygen and iron are drawn in red (small) and brown (large) spheres, respectively. The direction perpendicular to the surface is marked as (1$\bar{1}$02) in round brackets because there is no integer-valued vector in that direction. In the bulk, oxygen and iron are 4-fold and 6-fold coordinated, respectively; at the surface, they are 3-fold and 5-fold coordinated. (c, d) The same termination with adsorbed water in the configuration leading to the highest-temperature desorption peak (345 K, 2/3 of a monolayer).[2] Water is adsorbed in the form of half-dissociated dimers. Each water molecule and OH group is coordinated to one surface iron atom, and every third surface iron atom remains unoccupied at this coverage. O_{water} is drawn in blue, hydrogen in white.

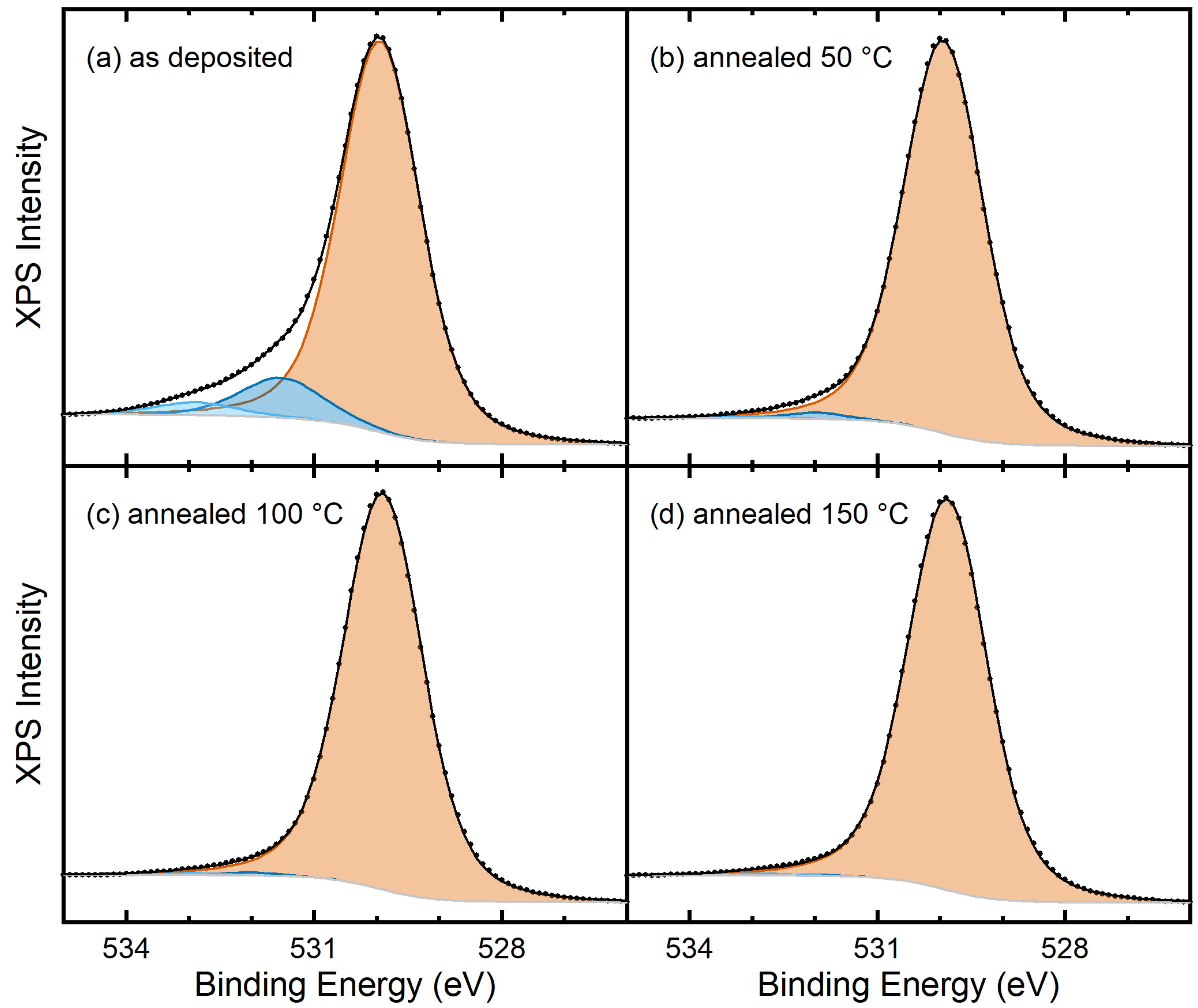

Figure S2: Fits of the O 1*s* region in XPS (Al Kα, 70° grazing emission, pass energy 16 eV) of 0.025 ML Rh stabilized by H_2O on α-$Fe_2O_3(1\bar{1}02)$ before and after annealing, also shown without fits in Figure 3(a). The data (black points) were fitted (solid lines) with a component corresponding to lattice O^{2-} anions at 529.9 eV (orange) and contributions from molecular H_2O (532.9 eV) and OH (531.5 eV).[2] The substrate peak shape has been optimized to match the pristine sample before Rh deposition. The contributions of the OH components to the overall peak areas are (a) 8.9%, (b) 1.2%, (c) 0.5%, (d) 0.2%. Errors due to data noise are negligible, but systematically larger OH contributions can be obtained if the as-prepared sample is assumed to already be hydroxylated.

Quantification of deposited rhodium

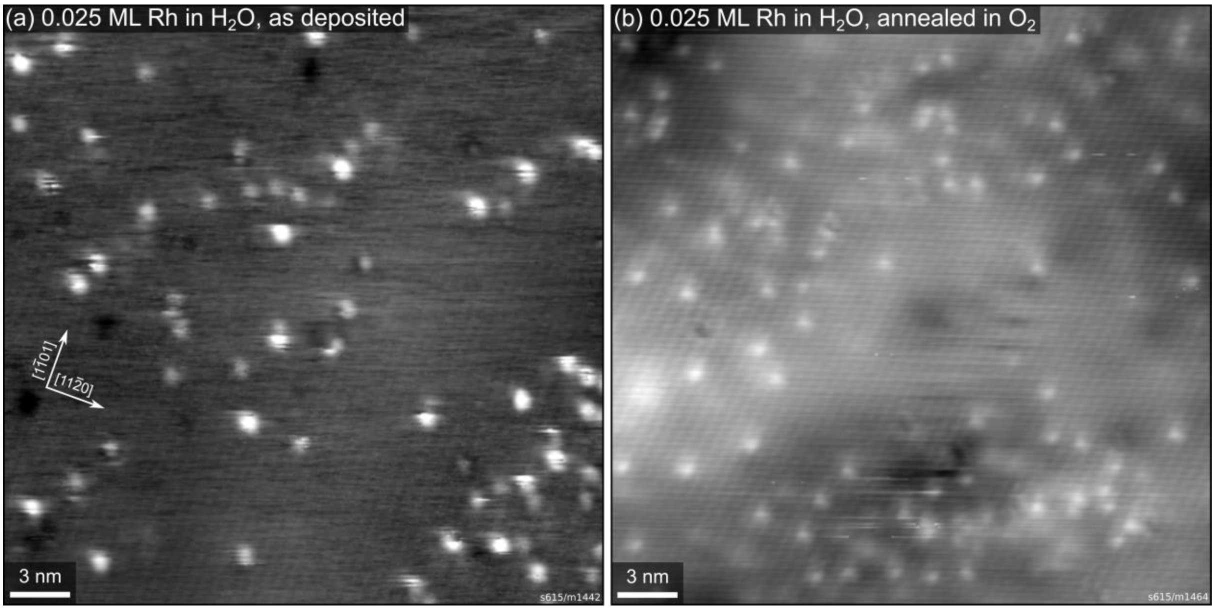

Figure S3: STM images of rhodium on α-$Fe_2O_3(1\bar{1}02)$ (a) after depositing a nominal dose of 0.025 ML Rh at room temperature in a background of 2×10^{-8} mbar H_2O (U_{sample} = +3.0 V, I_{tunnel} = 0.3 nA), and (b) after annealing the same sample at 520 °C in 2×10^{-6} mbar O_2 for 30 min (U_{sample} = −2.8 V, I_{tunnel} = 0.1 nA).

To determine whether the bright features found after Rh deposition in a background of water corresponded to single rhodium atoms, we analysed the area density after deposition, then annealed the sample at 520 °C in 2×10^{-6} mbar O_2 for 30 min to incorporate the Rh atoms. This results in well-defined, single Rh atoms substituting Fe in the first subsurface layer without significant loss of Rh to the bulk,[3] which allows us to easily quantify the coverage through STM analysis. STM images before and after incorporation are shown in Figure S3. The observed area densities are significantly lower than the nominal 0.025 ML (with 1 ML defined as two Rh atoms per unit cell, corresponding to a density of 7.3×10^{14} atoms/cm^{-2}), probably due to the calibration of the deposition rate with a quartz crystal microbalance being performed in UHV, while the actual deposition was performed in water background. However, the densities are approximately the same before and after incorporating the rhodium atoms into the surface, with 0.014 ML in Figure S3(a) and 0.015 ML in Figure S3(b). Therefore, we conclude that each bright feature in Figure S3(a) contains exactly one Rh atom.

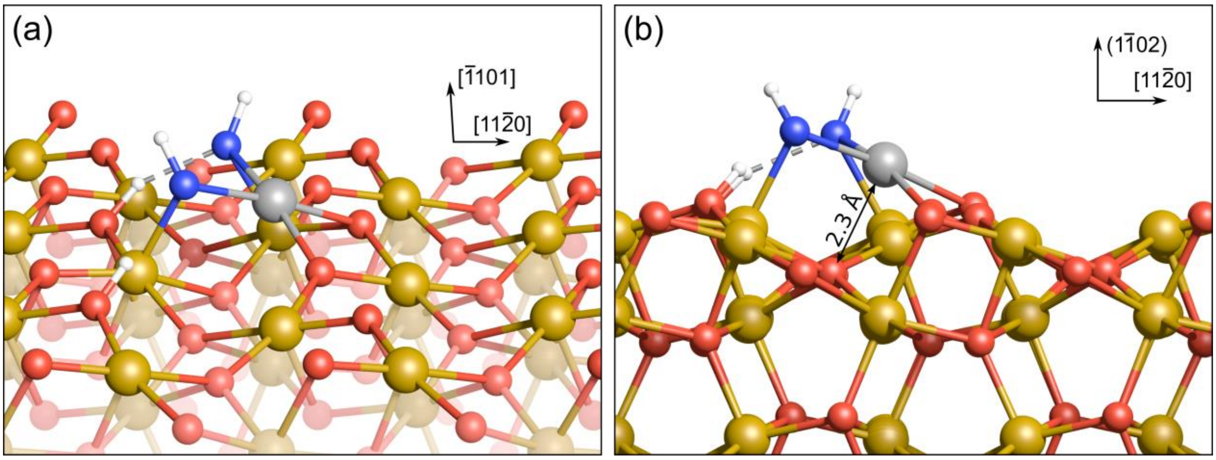

Figure S4: (a) Perspective and (b) side view of the schematic model of a $Rh(OH)_2$ complex shown in Figure 4(b). A Rh adatom (grey) is stabilized by two OH groups (O_{water} in blue, hydrogen in white). The Rh atom may also form an additional bond to a second-layer oxygen atom [distance in model as drawn marked in (b)], but that atom is already fully 4-fold coordinated in a bulk-like site.

1. Kraushofer, F.; Jakub, Z.; Bichler, M.; Hulva, J.; Drmota, P.; Weinold, M.; Schmid, M.; Setvin, M.; Diebold, U.; Blaha, P.; Parkinson, G. S., Atomic-Scale Structure of the Hematite α-Fe_2O_3(1$\bar{1}$02) "R-Cut" Surface. *J. Phys. Chem. C* **2018,** *122* (3), 1657-1669.
2. Jakub, Z.; Kraushofer, F.; Bichler, M.; Balajka, J.; Hulva, J.; Pavelec, J.; Sokolovic, I.; Mullner, M.; Setvin, M.; Schmid, M.; Diebold, U.; Blaha, P.; Parkinson, G. S., Partially Dissociated Water Dimers at the Water-Hematite Interface. *Acs Energy Lett* **2019,** *4* (2), 390-396.
3. Kraushofer, F.; Resch, N.; Eder, M.; Rafsanjani-Abbasi, A.; Tobisch, S.; Jakub, Z.; Franceschi, G.; Riva, M.; Meier, M.; Schmid, M.; Diebold, U.; Parkinson, G. S., Surface Reduction State Determines Stabilization and Incorporation of Rh on α-Fe_2O_3(1$\bar{1}$02). *Advanced Materials Interfaces* **2021,** *8* (8), 2001908.
